# The Availability of Iron Is Involved in the Murine Experimental *Toxoplasma gondii* Infection Outcome

**DOI:** 10.3390/microorganisms8040560

**Published:** 2020-04-14

**Authors:** Mário Cézar Oliveira, Loyane Bertagnolli Coutinho, Marcos Paulo Oliveira Almeida, Marisol Pallete Briceño, Ester Cristina Borges Araujo, Neide Maria Silva

**Affiliations:** Laboratory of Immunopathology, Institute of Biomedical Sciences, Federal University of Uberlândia, Uberlândia 38408-100, Brazil; cezar_cle@yahoo.com.br (M.C.O.); loyaneb.coutinho@ufu.br (L.B.C.); marcospaulooliveiraalmeida@hotmail.com (M.P.O.A.); marisolpb@gmail.com (M.P.B.); ester_borges@yahoo.com.br (E.C.B.A.)

**Keywords:** deferoxamine, dysbiosis, inflammation, iron, liver, lung, small intestine, *Toxoplasma gondii*

## Abstract

Iron is an important constituent of our environment, being necessary for both mammalian and pathogenic protozoa survival. Iron-containing proteins exert a wide range of biological processes such as biodegradation and biosynthesis, as well as immune function, fetal development, and physical and mental well-being. This work aimed to investigate the effect of iron deprivation in *Toxoplasma gondii* infection outcome. C57BL/6 mice were orally infected with *T. gondii* and treated with an iron chelator, deferoxamine, or supplemented with iron (ferrous sulfate), and the parasitism as well as immunological and histological parameters were analyzed. It was observed that the infection increased iron accumulation in the organs, as well as systemically, and deferoxamine treatment diminished the iron content in serum samples and intestine. The deferoxamine treatment decreased the parasitism and inflammatory alterations in the small intestine and lung. Additionally, they partially preserved the Paneth cells and decreased the intestinal dysbiosis. The ferrous sulfate supplementation, despite not significantly increasing the parasite load in the organs, increased the inflammatory alterations in the liver. Together, our results suggest that iron chelation, which is commonly used to treat iron overload, could be a promising medicine to control *T. gondii* proliferation, mainly in the small intestine, and consequently inflammation caused by infection.

## 1. Introduction

Iron is required as a functional component of many proteins in vertebrates, which are involved in a broad range of biochemical functions [1]. Therefore, iron acquisition is a fundamental requirement. In the presence of oxygen and at physiological pH, iron exists predominantly in the ferric Fe (Fe^3+^) form, which is insoluble, whereas iron transport systems take up the ferrous Fe (Fe^2+^), which is very instable and quickly oxidizes to ferric iron. Iron absorption occurs in the proximal duodenum by enterocytes in which ferric iron (Fe^3+^) is reduced by ferric reductase (such as duodenal cytochrome b, Dcytb) present in the apical brush border of enterocytes [2]. Ferrous iron (Fe^2+^) is then transported into the enterocytes mainly of the duodenum by the divalent metal ion transporter (DMT-1; formerly called DCT-1 or Nramp2), which is a member of the ‘natural-resistance-associated macrophage protein’ (Nramp) [3]. After transport into the enterocytes, ferrous iron can be used directly for intrinsic cellular metabolic processes (biosynthesis of heme), stored (intracellular ferritin), or exit the cell through the basolateral membrane transporter ferroportin (FPN1, or metal transporter protein 1 (MTP1) or iron-regulated transporter 1 (IREG1)), the mammalian cellular exporter of iron [4,5,6,7]. Ferroportin is localized on the surface of absorptive intestinal enterocytes, macrophages, hepatocytes, and placental cells, all of which release iron in plasma [4,5,6]. In mammals, the extracellular iron is transported by transferrin (TF) and lactoferrin (LF) [8,9].

A regulator of cellular iron homeostasis is the iron regulatory protein (IRP) system. When cellular iron levels are low, IRPs regulate the expression of numerous iron homeostasis proteins by binding to iron responsive elements (IREs); binding to 5′-untranslated regions inhibits the translation of ferritin, mitochondrial aconitase (ACO2), erythroid 5-aminolevulinate synthase, ferroportin 1, and enzyme 5-aminolevulinic acid synthase 2 (ALAS2). IRP binding to the 3′ IREs of transferrin-bound iron receptor (TfR) mRNA stabilizes its transcripts, resulting in increasing iron uptake [10,11]. At the systemic level, the iron hormone hepcidin is a major regulator of body iron balance. The hepcidin, a peptide hormone produced in the liver, regulates ferroportin and thus controls entry of iron into the plasma after enterocytes absorption; hepcidin induces the internalization and degradation of ferroportin in lysosomes [12]. In addition to hepcidin production in the liver, neutrophils and macrophages synthesize hepcidin in response to infectious agents, allowing for modulation of iron availability at the infectious focus [13].

Bacterial pathogens must acquire iron from their vertebrate hosts in order to replicate and cause disease [14], and protozoa parasites such as *Leishmania major* present *Leishmania* iron transporter (LIT1 and LIT2), which is essential for parasite replication within macrophage phagolysosomes [15,16]. *L. chagasi* have also developed mechanisms to utilize host transferrin and lactoferrin for growth [17]. For *Trichomonas vaginalis*, iron is important for parasite growth [18]. In rat enterocytes or in peritoneal macrophages, *Toxoplasma gondii* and *Trypanosoma cruzi* replication, respectively, are iron-dependent [19,20]; and it was demonstrated that two *T. gondii* membrane rhoptry proteins (ROP4 and ROP2) bind to human lactoferrin, which could be involved in parasite pathogenic mechanisms such as invasion and replication in the parasitophorous vacuole [21]. Our group showed that the addition of holo-transferrin increased, and deferoxamine (DFO) treatment decreased the *T. gondii* multiplication in human villous (BeWo) and in extravillous (HTR-8/SVneo) trophoblast cells, as well as in human chorionic villous explants [22]. Regarding another apicomplexan parasite, it was shown that iron chelators led to a dose-dependent inhibition of *Plasmodium* exoerythrocytic forms development in vitro and decreased liver infection in mice [23]. In acute *T. gondii* infection, deferoxamine, an iron chelator, induced 70% protection of Swiss mice against RH strain injection by the intraperitoneal route [24].

The main route of *T. gondii* infection in humans and other hosts is through ingestion of food or water that is contaminated with oocysts shed by cats or by eating undercooked or raw meat containing tissue cysts [25]. After oocyst or cysts ingestion, *T. gondii* proliferate in intestinal cells of mice [25,26]. In oral infection of C57BL/6 mice with low parasite load of the ME-49 strain, the parasite proliferates in the small intestine and disseminates to the other organs. In parallel, *T. gondii* provoked inflammatory alterations in the organs, mainly in the small intestine, lung, and liver in the acute phase of infection [26,27].

Considering the *T. gondii* iron demand for proliferation; the uptake of iron by the enterocytes, mainly the duodenum; and the fact that the parasite enters and proliferates in the intestinal cells before spreading to other organs, the aim of the present study was to investigate the effect of addition or deprivation of iron in parasite multiplication and infection outcome when the organism is administrated orally.

## 2. Materials and Methods

### 2.1. Animals

C57BL/6 female mice, 8–12 weeks old, were bred and maintained in the Bioterism Centre of the Animal Experimentation Laboratory, Biomedical Sciences Institute, Federal University of Uberlândia, MG, Brazil, with 12 h light/dark cycle and free access to food and filtered water. All experimental procedures were approved by the Animal Experimental Ethics Committee (CEUA) of the Federal University of Uberlândia, with protocol number 087/12, October, 31, 2012.

### 2.2. Parasites

The ME-49 strain of *T. gondii* was used to infect the animals in this study. The strain was maintained in chronically infected Swiss mice, which were inoculated with 10 cysts of *T. gondii* by oral route. A month after the inoculation, cysts were harvested from the brains and used to infect the experimental animals.

### 2.3. Experimental Design

C57BL/6 mice were injected intraperitoneally with ferrous sulfate heptahydrate (FeSO_4_) 100 mg/Kg to iron supplementation or deferoxamine (DFO) 300 mg/Kg, a chelator of iron, or with vehicle phosphate buffered saline (PBS), one day prior to oral infection with 20 ME-49 *T. gondii* cysts, and treated as described above for an additional seven days. Non-infected and untreated mice were analyzed as a control group. All reagents were purchased from Sigma Chemical Co., St Louis, USA. On day 8 post-infection, the animals were anesthetized with Ketamine (Syntec Brasil Ltd.a, Cotia, SP, Brazil) and Xylazine (Schering-Plough Coopers, Cotia, SP, Brazil) by intraperitoneal (i.p.) route and euthanized by cervical dislocation. Blood samples were collected by puncture of the retro orbital plexus for serological assays and tissue samples (small intestine, liver, and lungs) were collected, fixed in 10% buffered formalin, and processed routinely for paraffin embedding and sectioning or frozen immediately and stored in −80 °C for polymerase chain reaction (PCR) analysis.

### 2.4. Iron Staining in the Small Intestine, Lung, and Liver of Infected Mice

The iron accumulation in the small intestine was evaluated through Perls staining. Briefly, deparaffinized sections were incubated at room temperature with 10% of potassium ferrocyanide (Merck KGaA, Darmstadt, Germany) for 5 min and then incubated for 20 min with potassium ferrocyanide–10% hydrochloric acid solution (Merck S.A., Rio de Janeiro, RJ, Brazil). The sections were counterstained with nuclear rapid red (Merck KGaA, Darmstadt, Germany) and examined per tissue sections by light microscopy using a 40× objective. The images were analyzed using ImageJ software.

### 2.5. Iron Levels in Serum Samples

Iron serum levels were measured in non-hemolyzed serum samples by spectrophotometric analysis using a commercial kit (Fe Liquiform, Labtest Diagnóstica, Lagoa Santa, MG, Brazil). An external iron standard solution of concentration 223 µg/dL was employed.

### 2.6. Serum Levels of Pyruvic Transaminase

Serum levels of pyruvic transaminase (TGP) were analyzed by spectrophotometry in non-hemolyzed serum samples according to the manufacturer’s instructions (Labtest Diagnóstica S.A., Lagoa Santa, MG, Brazil). The absorbance was obtained at 505 nm.

### 2.7. Histological Analysis

For histological assay, tissue sections of the small intestine, lung, and liver were stained with Haematoxilin and Eosin and the analyses were done in two histological sections from each mouse in a blind manner. The inflammatory score was performed in the entire section of the small intestine as previously described [26] and was represented as arbitrary units: 0–2, mild; 2–4, moderate; 4–6, severe; and above 6, very severe. In the pulmonary tissue examined in a light microscope, the alveolar area was measured in 10 microscopic fields using the ImageJ software version 1.50i and, in the liver, the inflammatory foci were counted in 40 microscopy fields using a 10× objective. The Paneth cells were counted in 400 crypts in the small intestine section.

### 2.8. Immunohistochemical Analysis for Detection of Tissue Parasitism

The tissue parasitism was evaluated in the organs by immunohistochemistry as previously described [28]. Deparaffinized sections were incubated at room temperature with phosphate buffered saline plus 3% non-fat milk (Nestlé, São Paulo, Brazil) to reduce nonspecific binding, and then incubated at 4 °C overnight with polyclonal anti-*T. gondii* serum obtained from Swiss mice infected with ME-49 strain diluted in 0.01% saponin. After incubation with biotinylated goat anti-mouse antibody (Sigma Chemical Co., St. Louis, MO, USA), the assay sensitivity was improved by avidin–biotin–peroxidase complex (ABC kit, PK-4000; Vector Laboratories, Inc., Burlingame, CA, USA). The reaction was developed with 0.03% H_2_O_2_ plus 3,3′-diaminobenzidine tetrahydrochloride (DAB; Sigma) for 5 min. The sections were counterstained with Harris haematoxylin and examined under light microscope using a 40× objective. The tissue parasitism was scored by counting the number of cyst-like structures and parasitophorous vacuoles from two hundred microscopic fields in the small intestine, and in 40 microscopic fields in the lung or liver tissue section.

### 2.9. Relative Quantification of mRNA by qPCR

Small intestine sample mRNA was harvested using TRIzol reagent according to the manufacturer’s instructions (Life Technologies, Carlsbad, CA, USA). RNA concentration was determined (GeneQuant 1300 spectrophometer, GE Healthcare, Uppsala, Sweden) and complementary DNA (cDNA) was synthesized using 5 ng/mL mRNA through reverse transcription reaction following the manufacturer’s instructions (Promega, Madison, WI, USA). Quantitative PCR (qPCR) assays were performed using GoTAq^®^ qPCR (Master Mix, Promega, Madison, WI, USA) in Applied Biosystems 7500 Real-Time PCR System (Life Technologies). Assays were performed at 95 °C for 10 min and 40 cycles at 94 °C (1 min), 60 °C (30 s), and 72 °C (1 min). Specific primers used for murine were as follows: *gapdh*, F, 5′-GGAGAAACCTGCCAAGTATGATG-3′, and R, 5′-CAGTGTAGCCCAAGATGCCC-3′ [28]; *dmt1*, F, 5′-GCTCTGGGTGCTCCTCTT-3′, and R, 5′-CTTGGGATACTGACGGTGACA-3′; *dcytb*, F,5′-GGCTGCTGGTGTCCGC-3′, and R, 5′-CAGCCAAGCCCCTCTCG-3′, *ferroportin*, F, 5′-CTGTGTTTCTGGTGGAACTCTATGG-3′, and R, 5′-TCTTATCCACCCAGTCACCAATG -3′; and *hamp*, F, 5′-AGCCTGAGCAGCACCACCT-3′, and R, 5′- CAATGTCTGCCCTGCTTTCTT-3′. The unreferenced primers were designed using the Primer Express V3 software (Life Technologies).

### 2.10. Relative Quantification of Intestinal Bacteria by qPCR

The relative abundances of *Bacteroidetes*, *Firmicutes*, and *Proteobacteria* (family *Enterobacteriaceae*) were measured by qPCR using ABI PRISM-7500 sequence detection system (Applied Biosystems, Waltham, MA, USA) using GoTaq^®^ Probe qPCR Master Mix (Promega). The genomic DNA was extracted from the intestinal content (small intestine) using QIAamp DNA Stool Mini Kit (Quiagen). The specific primers for each bacterial population and for the 16S rRNA universal gene locus were as follows: *Firmicutes*, F, 5′-GGAGYATGTGGTTTAATTCGAAGCA -3′, and R, 5′-AGCTGACGACAACCATGCAC-3′ [29]; *Bacteroidetes*, F, 5′-CRAACAGGATTAGATACCCT-3′, and R, 5′-GGTAAGGTTCCTCGCGTAT-3′ [29]; *Enterobacteriaceae*, F, 5′-GTGCCAGCMGCCGCGGTAA-3′, and R, 5′-GCCTCAAGGGCACAACCTCCAAG-3′ [30]; *E. coli*, F, 5′-AAGACGTATTCTCCATCTCCGG-3′, R, 5′-ACGGCCGCCTTCATCTTTGGACAG-3′ [31]; and 16S rRNA universal, F, 5′-ACTCCTACGGGAGGCAGCAG-3′, R, 5′-ATTACCGCGGCTGCTGG-3′ [29]. Cycling conditions were performed at 95 ° C for 10 min and 40 cycles at 95 °C (15 s), 60 °C (1 min), and 60 °C (1 min).

### 2.11. Cytometric Bead Array Assay

The cytokines interleukin (IL)-6, IL-10, tumor necrosis factor (TNF), and interferon (IFN)-γ were quantified using a cytometric bead array assay (CBA) (BD, San Jose, CA, USA) according to the manufacturer’s instructions. Samples were acquired using a FACSCanto II Flow Cytometer (BD, Biosciences, México) and analyzed with FACSDiva software (BD).

### 2.12. Statistical Analysis

The data were analyzed using GraphPad Prism 8 software (GraphPad Software Inc., San Diego, CA, USA, EUA). Data were expressed as mean ± SEM of experimental groups. The differences between groups were analyzed by Kruskal Wallis test or analysis of variance (ANOVA) followed by Dunn or Sidak’s post-test, respectively, when appropriate. Additionally, comparisons between two experimental groups were performed with the Mann–Whitney unpaired test. Differences were considered statistically significant when *p* < 0.05.

## 3. Results

### 3.1. Toxoplasma gondii Infection Induces Iron Accumulation in the Organs of Infected Animals

**Figure 1:**
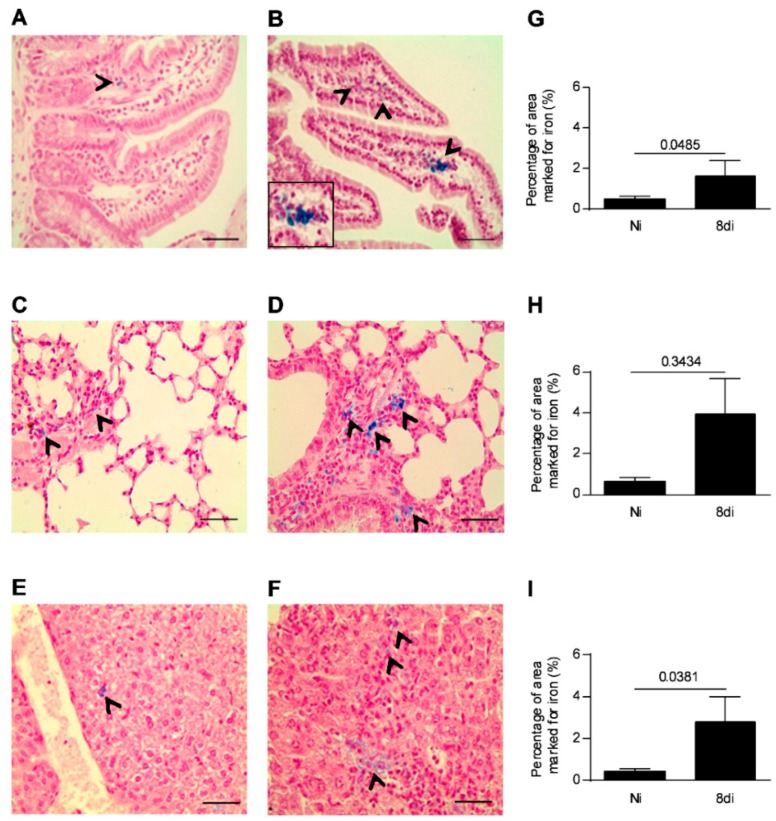
Iron positive areas in the small intestine, lung, and liver of *T. gondii*-infected mice. Representative photomicrographs of iron positive areas detected by Perls staining in the small intestine (**A**,**B**), lungs (**C**,**D**), and liver (**E**,**F**) of C57BL/6 female mice infected with 20 cysts of ME-49 *T. gondii* strain or non-infected mice examined on day 8 of infection. The areas with cells iron stained (blue) were captured per tissue sections by light microscopy using 40× objective and quantified using ImageJ software (**G**–**I**). Non-infected (Ni); days of infection (di). The black arrows indicate areas with iron positive cells. Mann–Whitney test. Scale bar = 50 µm.

As the parasite load in the small intestine is higher after five days of infection [32], we investigated if *T. gondii* infection is able to induce iron accumulation in the small intestine as a necessity for its replication and proliferation on day 8 of parasite inoculation. In addition, the iron levels were also investigated in the pulmonary and hepatic tissues, as these organs are also parasitized in this phase of infection. It was observed that infected mice showed higher iron accumulation in the small intestine, lung, and liver (Figure 1B,D,F) on day 8 of parasite inoculation compared with non-infected mice (Figure 1A,C,E). The iron augmentation was significantly higher in the small intestine and liver of infected mice than in those of non-infected mice (*p* = 0.0485 and *p* = 0.0381, respectively; Figure 1G,I), and showed a trend to be higher in the lung of infected mice (Figure 1H), suggesting that increased iron accumulation in the organs induced by *T. gondii* infection could be involved in the nutritional need for iron by the parasite.

### 3.2. Infection with T. gondii Interferes in the Expression Genes Involved in the Iron Absorption by the Small Intestine

**Figure 2:**
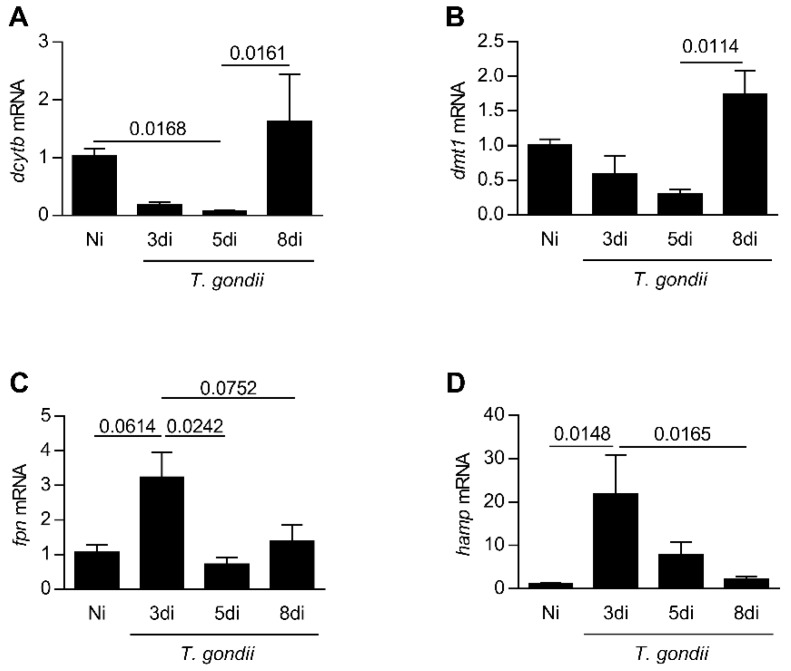
Expression levels of genes related to iron absorption in the small intestine in a kinetics days of *T. gondii* infection. Quantification of messages by quantitative PCR reaction (qPCR) for *dcytb* (**A**), *dmt1* (**B**), *fpn* (**C**), and *hamp* (**D**) in the small intestine from C57BL/6 mice infected by the oral route with *T. gondii* ME-49 strain. Non-infected (Ni); days of infection (di). Relative expression: expression of the indicated gene in relation to the *gapdh* gene. The data are representative of two independent experiments (*n* = 4). Data were analyzed by Kruskal Wallis followed by Dunn’s post-test (**A**,**B**,**D**) and one-way analysis of variance (ANOVA) followed by Sidak’s post-test (**C**).

In the next step, the expression levels of genes related to iron absorption in the small intestine in a kinetics of days of infection were analyzed. It was observed that the ferric reductase, *dcytb*, decreased from day 3 to 5 (*p* = 0.0168) of infection, and *dmt1* presented a trend to diminish until day 5 post-infection; both genes are involved in the iron absorption (Figure 2A,B). The *Ferroportin* (*fpn*) mRNA expression showed a trend to be higher on day 3 of infection (*p* = 0.0614) in relation to non-infected mice. Interestingly, an increase in the *hamp* mRNA expression (*p* = 0.0148) was detected on day 3 of infection (Figure 2C,D). On day 8 of infection, the expression levels of these genes were similar to those observed in non-infected animals (Figure 2A–D).

### 3.3. T. gondii Infection Increases the Iron Concentration Systemically on Day 8 of Infection

**Figure 3:**
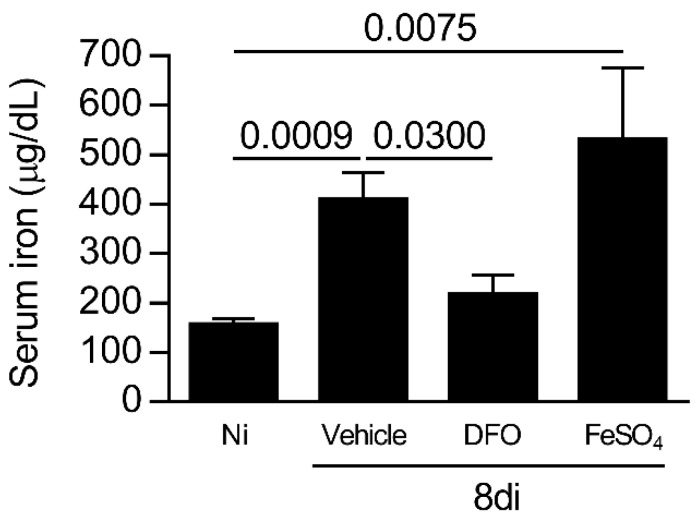
Iron levels in the sera of C57BL/6 *T. gondii*-infected mice treated with an iron chelator or iron supplemented. Iron serum levels of C57BL/6 mice non-infected (Ni) or infected with 20 cysts of ME-49 strain of *T. gondii* by the oral route and treated with vehicle (PBS, phosphate buffered saline) or 300 mg/kg deferoxamine (DFO) or 100 mg/kg of ferrous sulfate heptahydrate (FeSO_4_) intraperitoneally (i.p.) one day before infection and for an additional seven days post-infection. Data were analyzed by Kruskal Wallis followed by Dunn’s post-test.

To evaluate the involvement of iron in parasite growth during oral *T. gondii* infection, C57BL/6 mice were infected and treated with DFO (iron chelator) or supplemented with iron, sulfate heptahydrate (FeSO_4_), and the systemic iron levels were measured. It was observed that *T. gondii*-infected mice treated with FeSO_4_ or vehicle presented higher iron serum levels compared with non-infected mice (*p* = 0.0009 and 0.0075, respectively), and DFO treatment was able to decrease the iron levels systemically (*p* = 0.0300; Figure 3). Thus, the mortality was accompanied in infected treated mice, and it was observed that the treatment of *T. gondii*-infected mice with DFO or FeSO_4_ did not alter the survival rate of the animals.

### 3.4. Iron Availability is Involved in the Parasite Growth and Inflammation in the Small Intestine

**Figure 4:**
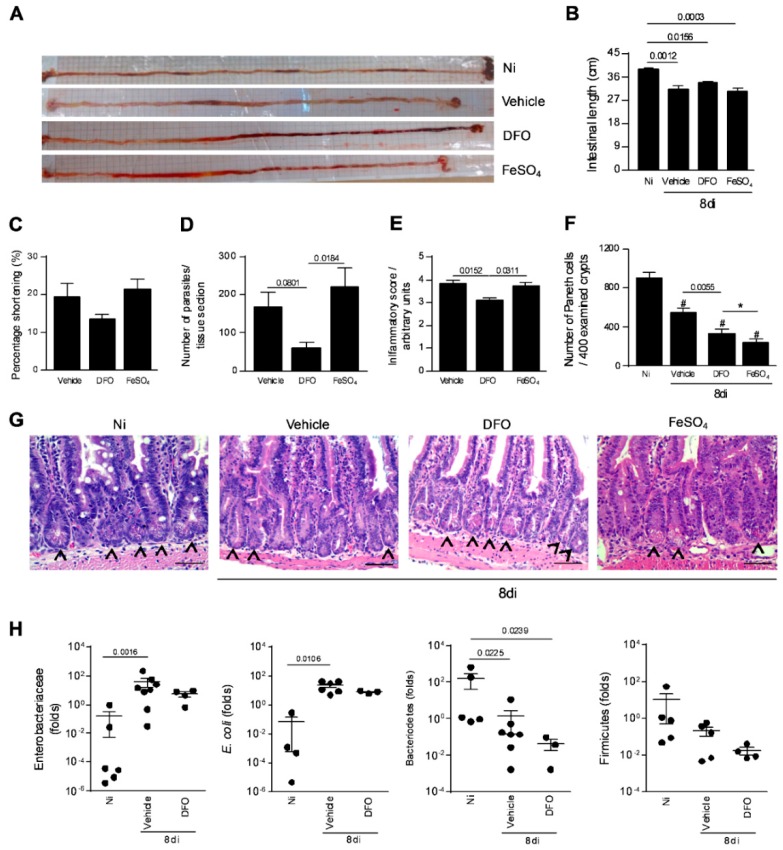
Small intestine parasitism, inflammatory alterations, and relative quantification of some intestinal bacterial population in *T. gondii*-infected mice treated with an iron chelator or iron supplemented. Gross picture of the small intestine of non-infected (Ni) or infected C57BL/6 mice with 20 ME-49 *T. gondii* cysts by oral route and treated with vehicle (PBS) or 300 mg/Kg of deferoxamine (DFO) or 100 mg/Kg of iron sulfate heptahydrate (FeSO_4_) by intraperitoneal (i.p.) injection one day prior to infection, and for an additional seven days post-infection (**A**). Intestinal length (**B**) and shortening percentage of the small intestine (**C**). Tissue parasitism in the small intestine was detected by immunostaining and quantified by tissue section (**D**). Inflammatory score in the small intestine (**E**). Quantification of Paneth cells in 400 intestinal crypts (**F**,**G**). Arrows indicate Paneth cells. Relative quantification of intestinal *Enterobacteriaceae*, *E. coli*, *Bacteriodetes*, and *Firmicutes* by qPCR (**H**). Data were analyzed by one-way analysis of variance (ANOVA) followed by Sidak’s post-test (**E**,**F**) and Kruskal Wallis followed by Dunn’s post-test (B, D, H). (*)(#) *p* < 0.0001; (#) differences in relation to non-infected (Ni) mice. Scale bar = 50 µm.

It was previously shown that *T. gondii* infection induces intestinal shortening [33]. In the present investigation, it was verified that vehicle-treated infected (*p* = 0.0012), FeSO_4_ supplemented infected (*p* = 0.0003), and DFO-treated infected mice (*p* = 0.0156) showed significantly lower intestinal length compared with non-infected and untreated mice (Figure 4A,B). Analyzing the intestinal shortening percentage, this parameter presented a trend to be lower in DFO-treated in comparison with infected vehicle-treated or FeSO_4_ supplemented mice (Figure 4C). With regard to tissue parasitism, the decrease in iron availability induced by DFO treatment decreased the parasite load in the small intestine compared with mice treated with vehicle (*p* = 0.0366), whereas FeSO_4_ supplementation increased the parasite load in relation to DFO-treated infected mice (*p* = 0.0138), and showed a trend to increase the parasite load compared with vehicle-treated infected mice (Figure 4D). When analyzing the iron accumulation in the organ, it was observed that DFO-treated animals presented lower iron detection by Perls staining (Supplementary Figure S1).

Accordingly, DFO-treated mice showed a significantly lower inflammatory score in the small intestine when compared with both vehicle-treated (*p* = 0.0152) or FeSO_4_ supplemented mice (*p* = 0.0311) (Figure 4E).

As oral infection with *T. gondii* leads to elimination of Paneth cells and dysbiosis [34], the Paneth cell numbers in the small intestine of infected mice treated with DFO or FeSO_4_ were verified. It was observed that the number of Paneth cells per 400 examined crypts decreased in *T. gondii*-infected mice (*p* < 0.0001). Interestingly, when animals were DFO-treated, they at least partially preserved the Paneth cell numbers, with the cell phenotype number being higher compared with animals infected treated (*p* = 0.0055) and infected FeSO_4_-supplemented (*p* < 0.0001) (Figure 4F,G). As DFO partially preserved the Paneth cell numbers under infection, the relative abundance of *Bacteroidetes*, *Firmicutes*, and *Enterobacteriaceae* when animals are treated with DFO and infected with *T. gondii* was investigated. In *T. gondii*-infected mice supplemented or not with FeSO_4_, an expansion of *Enterobacteriaceae*, such as *E. coli* on day 8 after *T. gondii* inoculation, as well as a simultaneous loss of *Bacteroidetes*, were verified, while the relative abundance of *Firmicutes* remained unchanged. The infected DFO-treated mice also presented a decrease in the *Bacteroidetes* population, however, the *Enterobacteriaceae* population did not increase significantly (Figure 4H).

These results suggest that iron is required for *T. gondii* replication in the small intestine during oral infection. Additionally, the decrease in parasite replication owing to DFO treatment leads to a low inflammatory alteration in the small intestine, less pronounced Paneth cell loss, and lower dysbiosis.

### 3.5. Iron Availability Is Involved in the T. gondii Proliferation and Spread to the Other Organs

**Figure 5:**
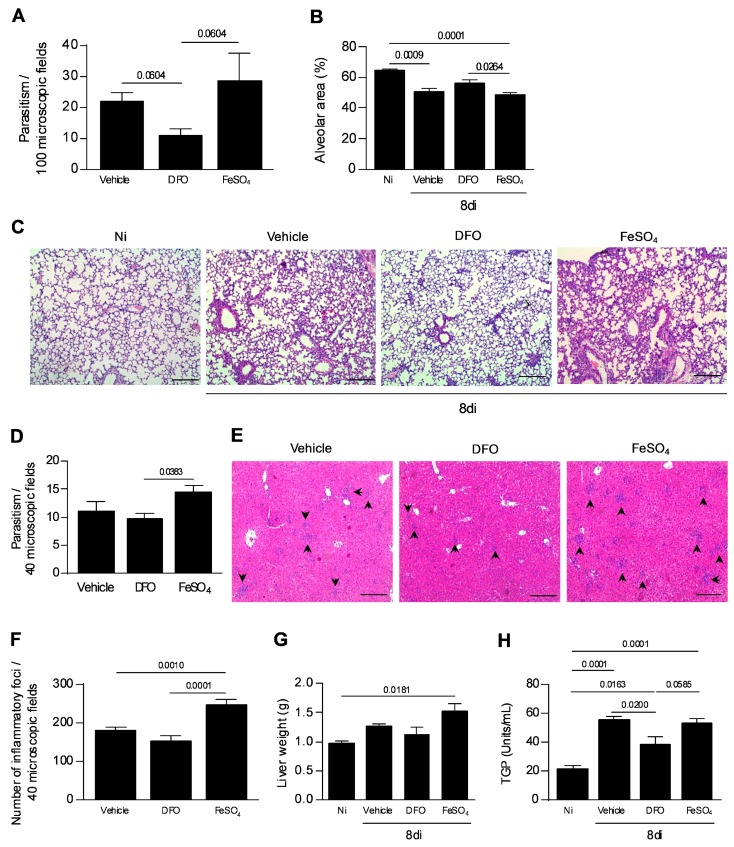
Tissue parasitism and inflammatory alterations in the lung and liver of *T. gondii*-infected mice treated with an iron chelator or iron supplemented. C57BL/6 mice were orally infected with 20 cysts of the *T. gondii* ME-49 strain and treated with vehicle (PBS) or 300 mg/kg of deferoxamine (DFO) or 100 mg/kg of ferrous sulfate heptahydrate (FeSO_4_) by intraperitoneal (i.p.) route one day before infection, and for an additional seven days after infection. The parasitism was quantified in 100 microscopic fields (**A**). The measurement of the alveolar area in 10 microscopic fields (**B**). Representative photomicrographs of pulmonary tissues (**C**). Tissue parasitism in the liver in 40 microscopic fields (**D**). Quantification of inflammatory foci in the liver in 40 microscopic fields (**E**). Representative photomicrographs of the inflammatory foci in the liver (**F**). The arrows indicate the inflammatory foci. Liver weight (**G**). Serum levels of pyruvic transaminase (TGP) (**H**). Non-infected (Ni); days of infection (di). Data were analyzed by Kruskal Wallis followed by Dunn’s post-test (**A**,**H**) and one-way ANOVA followed by Sidak’s post-test (**B**,**G**,**H**). Scale bar = 50 µm.

As the presence of iron is required by the parasite proliferation, the next step was to analyze whether *T. gondii* spread to the other organs would be altered by iron deprivation. It was observed that the parasite burden found in the lung of vehicle or FeSO_4_-treated mice showed a tendency to be greater than that observed in DFO-treated mice (Figure 5A). In parallel, the alveolar area was measured as indicative of lung function. It was observed that the alveolar area of DFO-treated mice was similar to that of non-infected and higher than that of FeSO_4_-treated mice (*p* = 0.0264) (Figure 5B,C). In the liver, iron supplementation induced an increase in tissue parasitism compared with DFO (*p* = 0.0383) (Figure 5D). In addition, FeSO_4_ supplementation induced a significant increase in the number of inflammatory foci in the organ compared with non-treated mice (*p* = 0.0010) or DFO treatment (*p* ≤ 0.0001). DFO treatment apparently decreased the inflammation in the liver (Figure 5E,F). In parallel, a higher weight liver from FeSO_4_-treated mice (Figure 5G) was detected in comparison with noninfected mice, and TGP measurement showed elevated levels in all infected mice; however, DFO treatment decreased the TGP levels in serum samples of infected mice compared with vehicle-treated mice and showed a trend to decrease in relation to FeSO_4_-treated mice (*p* = 0.0200, *p*= 0.0585, respectively) (Figure 5H).

**Figure 6:**
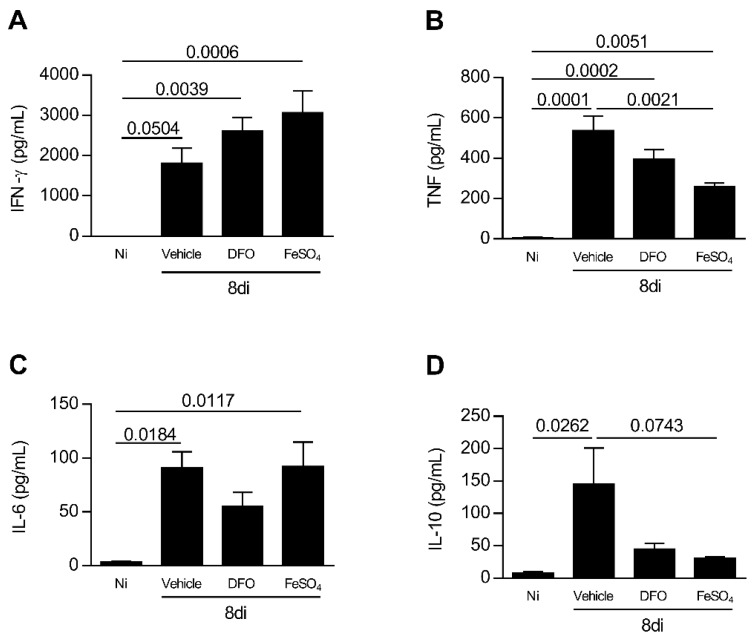
Systemic cytokine profile of *T. gondii*-infected mice treated with an iron chelator or iron supplemented. C57BL/6 mice were orally infected with 20 *T. gondii* ME-49 cysts and treated with vehicle (PBS) or 300 mg/kg deferoxamine (DFO) or 100 mg/kg of ferrous sulfate heptahydrate (FeSO_4_) intraperitoneally (i.p.) one day before infection and for an additional seven days after infection. The levels of IFN-γ (**A**), TNF (**B**), IL-6 (**C**), and IL-10 (**D**) were measured by the CBA method (Cytometric bead array). Data were analyzed by one-way ANOVA followed by Sidak’s post-test (**A**–**D**) and Kruskal Wallis followed by Dunn’s post-test.

*T. gondii* infection induces Th1 profile of immune response [35]. To verify whether iron availability or deprivation affects the cytokine profile in *T. gondii* infection, the levels of IFN-γ, TNF, IL-6, and IL-10 were measured in serum samples of experimental groups of mice. The infection with *T. gondii* increased the IFN-γ and TNF levels systemically, irrespective of whether or not the animals were treated with DFO or FeSO_4_ (Figure 6A,B). The infection also increased the IL-6 levels in serum samples of animals non-treated and treated with FeSO_4_, and showed a trend of being higher in infected DFO-treated animals (Figure 6C). Animals supplemented with FeSO_4_ presented a decrease in TNF levels in serum samples in comparison with vehicle treatment (*p* = 0.0021) (Figure 6B), however, the levels of IL-10 presented a trend to be smaller in comparison with vehicle-treated mice (*p* = 0.0743). In addition, the levels of IFN-γ were apparently higher in FeSO_4_-treated mice compared with vehicle-treated mice (Figure 6A).

When analyzing IL-10 in serum samples, it was observed that infection with the parasite increased IL-10 levels systemically (*p* = 0.0262), and mice infected and treated with DFO or FeSO_4_ apparently produced more IL-10 compared with non-infected mice (Figure 6D).

## 4. Discussion

The host–pathogen interaction allows the parasite to compete with its hosts for acquisition of many essential compounds, such as glucose, cholesterol, tryptophan, and iron [15,16,36,37,38]. Parasitic infectious organisms such as *T. brucei* [39], *T. gondii* [21], and *Trichomonas vaginalis* [40] present lactoferrin binding protein in their surface, and lactoferrin [40,41] or transferrin [42] is utilized by *Entamoeba histolytica* and *Trichomonas vaginalis* or *T. brucei*, respectively, for their growth; furthermore, both transferrin and lactoferrin are used for promastigotes of *L. chagasi* proliferation [17]. During oral transmission, tachyzoites of *T. gondii* develop within the intestinal epithelium through a fast growth, which lyse their cells and releases large numbers of progeny, indicating a high need for nutrients [43]. In accordance, many enzymes associated with the acquisition of host nutrients such as glucose and cholesterol are up-regulated during infection of human foreskin fibroblast (HFF) cells with *T. gondii* [44]. In the present study, when C57BL/6 mice were orally infected with *T. gondii*, an accumulation of iron in the intestinal epithelium, lung, and liver was observed on day 8 of the infection, as well as an increase of iron serum levels, suggesting that iron is a necessary nutrient for the development and proliferation of *T. gondii*. Interestingly, the transferrin receptor (TfR) was found to be specifically up-regulated in *Toxoplasma*-infected HFF, the function of which is iron uptake through binding of transferrin [45,46]. Additionally, Dziadek’s group identified two rhoptry protein families (ROP2 and ROP4) in *T. gondii* that are capable of binding to the iron transporting protein, the lactoferrin [21,47]. Thus, for the first time, the mRNA of genes involved in iron metabolism was measured in the small intestine of mice infected by the oral route with *T. gondii*. It is known that hepcidin is induced in acute inflammation. C57BL/6 mice challenged with sublethal doses of lipopolysaccharide (LPS) increased hepcidin mRNA levels in the liver after 6 h, in parallel with a decrease in the mRNA and protein expression levels of ferroportin in the duodenum [48]. In rats, hepatic increased hepcidin levels were detected in the acute phase response, peaking at 8 h following Freund’s complete adjuvant intraperitoneal injection [49]. Additionally, hepcidin represses iron absorption as mice lacking the hepcidin gene accumulate excess iron [50]. Although hepatocytes are the main source of hepcidin [51], in the present investigation, it was observed that hepcidin mRNA expression in the small intestine enhanced 19-fold on day 3 of *T. gondii* inoculation, suggesting that the hepcidin could alter the iron availability to the parasite at the foci of infection precociously. Interestingly, in accordance with previous studies that observed an inverse relationship between the hepcidin expression, that increases in the liver, and Dcytb and DMT1 that decrease in the duodenum in acute inflammation [48,49], we observed a significant decrease in the small intestine Dcytb mRNA mainly on day 5 of infection, suggesting repression in the iron absorption. As ferroportin is localized on the surface of absorptive intestinal enterocytes and hepcidin induces the internalization and degradation of ferroportin [12], we detected an apparent increase in the ferroportin expression on day 3 of infection. This could suggest a compensatory mechanism to ferroportin degradation induced by hepcidin increase, as hepcidin mRNA expression was augmented, suggesting an increase of protein expression levels. The hepcidin increase and Dcytb decrease mRNA expression levels, suggesting that it could be a host-defense mechanism trying to retain the parasite proliferation in a more precocious phase of parasite entry; however, as the infection progress, the parasite itself could be involved in the return of the expression of these genes to levels of non-infected animals, for its own benefit. On the other hand, hepcidin enhanced expression levels could induce ferroportin degradation in the enterocytes, favoring iron accumulation inside the cell, and consequently *T. gondii* growth and infection progress. Additional experiments are necessary to clarify these points.

Deferoxamine mesylate is a hexadentate iron chelator with antioxidant properties [52]. Iron overload catalyzes the formation of highly reactive hydroxyl radicals, which causes membrane damage and protein denaturation, and deferoxamine is used in iron chelation [53]. Many in vivo and in vitro studies have demonstrated that deferoxamine has beneficial effects in the treatment of protozoan infections, such as *T. cruzi* and *P. berghei*, being free of major hematological side effects [54,55,56]. As intestinal rat [20], human foreskin fibroblast (HFF) [46], and human trophoblast [22] cells treated with deferoxamine prevented *T. gondii* replication in a dose-dependent manner, we treated C57BL/6 mice by the oral route with deferoxamine and observed the infection outcome, mainly the parasite growth and lesions in the small intestine, lung, and liver in acute phase of infection. In the present investigation, it was observed that deferoxamine inhibited the growth of *T. gondii* in the small intestine. The lower parasite load observed in the organ could be related to the iron deprivation for the parasite growth. Thus, as observed in vitro for the RH strain [22,46], the iron deprivation also is able to interfere in the ME-49 *T. gondii* strain growth in vivo. However, with our experimental protocol, the iron supplementation of animals with FeSO_4_ was not able to increase the tissue parasitism in levels higher than those of infected untreated mice. Increased parasitemia related to iron availability has been seen in in vivo and in vitro studies with *Plasmodium*. Culture of Huh7 hepatoma cells infected with *Plasmodium berghei* presented a higher percentage of the exoerythrocytic area after iron supplementation with ferric ammonium citrate, as well as a higher liver parasite load of *P. berghei* sporozoite-infected mice 40 h after iron supplementation [23]. Additionally, iron supplementation with ferrous sulfate reversed the cobalt protoporphyrin-mediated decrease in parasitism in peritoneal macrophages infected in vitro with *T. cruzi* [56]. Thus, the different results obtained in our investigation could be related to the iron source in association with the parasite species. In addition, it was verified that DFO treatment ameliorates the intestinal parameters that are affected by *T. gondii* infection such as length and inflammatory changes of the small intestine. These beneficial effects of DFO treatment could be related to the decrease of the parasite load, as well as the antioxidant properties of the drug.

Paneth is a specialized cell phenotype in the small intestine that produces α-defensin, lysozyme, C-type lectins, and phospholipase A2 (PLA2), which are antimicrobial proteins important to maintain intestinal homeostasis [57]. These cells are depleted from the small intestine of C57BL/6 mice inoculated by the oral route with ME-49 strain of *T. gondii* on day 7 of infection, in an IFN-γ-dependent manner [34]. Production of antimicrobial peptides by Paneth cells is able to protect against enteric bacterial pathogens [58]. The dysbiosis with uncontrolled expansion of the *Enterobacteriaceae* family of Gram-negative bacteria occurred in association with the Paneth cell loss in *T. gondii* infection [34]. Additionally, the *T. gondii*-induced dysbiosis characterized by expansion of *Enterobacteriaceae* contributed to intestinal pathology, as germ-free mice inoculated with *T. gondii*, adoptively transferred with *Enterobacteriaceae* isolated from *T. gondii*-infected mice, developed a similar intestinal pathology [34]. Herein, in accordance with previous studies [34,59,60], we observed that the infection with *T. gondii* significantly decreased the Paneth cell numbers in the small intestine, in parallel with an increase in the *Enterobacteriaceae* family of bacteria. When animals were treated with DFO, they preserved at least in part the Paneth cell numbers and in parallel did not present a significant increase in the *Enterobacteriaceae* family of bacteria, suggesting that the presence of Paneth cells limited the expansion of the *Enterobacteriaceae* family and decreased the intestinal pathology.

After oral ingestion, *T. gondii* crosses the intestinal epithelium and disseminates to the deep tissues [27]. *T. gondii* enters hosts through the intestinal mucosa and infected CD11c- and CD11b-expressing mouse leukocytes, transporting the parasite to colonize distant tissues such as the brain [61]. Here, we showed that *T. gondii* migrates to the lung and liver, as was observed the parasite in these organs on day 8 p.i., and the DFO treatment presented a tendency to diminish the parasite growth in the lung and, in parallel, preserved the alveolar area, improving the lung function. Additionally, iron supplementation apparently increased the parasite growth in the liver in relation to non-treated infected mice, and significantly in relation to DFO-treated mice, in parallel with higher inflammatory alterations. The liver is an organ that storage the excess of body iron [62], and thus could be an important organ for the development of the parasite under iron supplementation. In our investigation, we expected to find a massive *T. gondii* growth in the organ of FeSO_4_-treated mice; however, in disagreement with our prediction, we did not detect an intense augmentation of parasite growth in the organ of supplemented mice. It could be the iron source utilized in our experimental procedure, as it was previously shown that the ferric ammonium citrate, a source of iron supplementation, increased the *P. berghei* parasite load in the liver [23]. Under infection, *T. gondii* triggers IL-12 production, which acts with TNF to induce IFN-γ by natural killer (NK) cells; being that the infection normally triggers protective cell-mediated immunity, in which IFN-γ has a central role [63]. IFN-γ and TNF are important in the induction of reactive nitrogen intermediates, such as nitric oxide (NO), that present microbicidal activity against tachyzoites [64,65]. However, when certain mouse lineages are infected by the oral route, such as C57BL/6, an exacerbated Th1 immune response develops with massive necrosis of the epithelial cells of the small intestine, with CD4^+^ T cells, IFN-γ, TNF, and nitric oxide (NO) mediating the development of intestinal lesions [66,67]. In our present study, it was observed that the amount of iron availability for *T. gondii* metabolism did not alter the increased IFN-γ and TNF levels that normally occur in *T. gondii* infection. In addition, Okada’s group demonstrated increased IFN-γ receptor expression, IFN-γR1, and mainly IFN-γR2 in the hepatocellular carcinoma cell line after treatment with DFO [68], suggesting that IFN-γ could more efficiently perform its protective mechanism under DFO treatment.

Interleukin-6 (IL-6) is a cytokine with pleiotropic activities, being produced by many different cell types including monocytes/macrophages, fibroblasts, keratinocytes, endothelial cells, mesangial cells, glial cells, chondrocytes, osteoblasts, smooth muscle cells, T cells, B cells, granulocytes, mast cells, and certain tumor cells [69]. IL-6^-/-^ 129/Sv C57BL/6 backgrounds were more susceptible than those of their control mice when infected with 10 cysts by the intraperitoneal route, presenting large areas of necrosis with presence of tachyzoites. Animals presented greater numbers of cysts than control mice, and small amounts of IFN-γ and higher IL-10 mRNA levels in the brain at week 8 of infection [70]. In accordance, when IL-6^-/-^ 129/SvJ mice were infected with RRA strain by the oral route with 20 cysts, they were more susceptible, with high mortality until 28 days p.i. and high parasite load with high IFN-γ serum levels on day 7–8 of infection, with high inflammation in the brain with necrotic areas [71]. However, the amounts of IL-6 must be well controlled, as C57BL/6 that lack SOCS3 in neutrophil and macrophage lineages infected with 10^4^ Prugniaud tachyzoites by intraperitoneal route were highly susceptible to *T. gondii* dying until 10 days p.i., and presented increased IL-6 production at day 8 p.i.; the susceptibility was associated with higher IL-6 levels, which becomes a potent antagonist of IL-12 production [72]. Our data showed that deferoxamine treatment decreased 1.65-fold the IL-6 serum levels in relation to untreated infected mice. IL-6 can act in the systemic regulation of iron through the induction of the hormone hepcidin and hepcidin regulates the entry of iron into the bloodstream, imprisoning the iron inside the enterocytes [12]. Thus, IL-6 must be fine-tuned, as high IL-6 levels induced by oral *T. gondii* infection could be beneficial for the parasite growth, as the hepcidin-IL-6 axis increases the availability of iron within the enterocytes, enabling the growth of the parasite. The anti-inflammatory cytokine IL-10 plays a critical role in the control of inflammatory responses to *T. gondii* infection. IL-10^-/-^ C57BL/10 mice are unable to counteract the inflammatory effects of IFN-γ induced by the infection with the ME-49 strain by the intraperitoneal route, and succumb during the acute stage of the disease with enhanced liver pathology and IL-12 and IFN-γ production [73]. In our study, despite not being statistically significant, the IL-10 production was increased in *T. gondii* infected mice, and DFO treatment did not significantly alter its levels.

## 5. Conclusions

In conclusion, the present work showed that iron is an important nutrient for *T. gondii* proliferation, mainly in the small intestine in oral infection, and the decreased parasite load, owing to the lower availability of iron, partially preserves the Paneth cell numbers and diminishes the *Enterobacteriaceae*, ameliorating the intestinal inflammation.

## Supplementary Materials

The following are available online at https://www.mdpi.com/2076-2607/8/4/560/s1.
