# Supplementary material for: The Availability of Iron Is Involved in the Murine Experimental Toxoplasma gondii Infection Outcome
Source: Microorganisms. 2020 Apr 14;8(4):560. doi: 10.3390/microorganisms8040560 (PMC7232304; doi:10.3390/microorganisms8040560)
Supplement: Supplementary file 1 [file microorganisms-08-00560-s001.pdf]

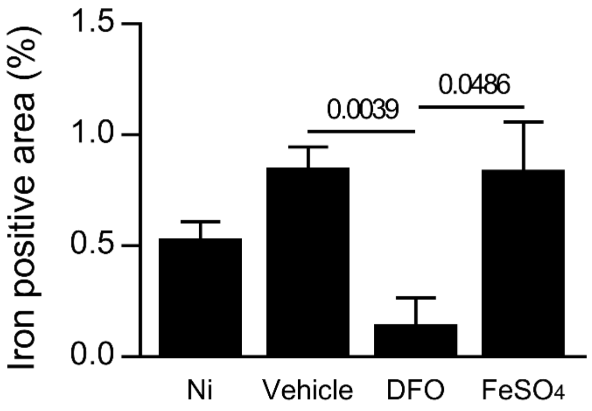

Supplementary Figure S1. Iron positive areas in the small intestine of *T. gondii*-infected mice treated with DFO or $FeSO_4$. C57BL/6 mice were infected with 20 cysts of ME-49 *T. gondii* strain by oral route and treated with vehicle (PBS) or 300 mg/Kg of deferoxamine (DFO) or 100 mg/Kg of iron sulfate heptahydrate (FeSO4) by intraperitoneal (i.p.) injection one day prior infection and for additional 7 days post-infection. The iron in the intestinal tissue section was detected by Perls staining and the areas with cells iron stained (blue) were captured per tissue sections by light microscopy using 40x objective and quantified using ImageJ software. Data were analyzed by Kruskal Wallis followed by Dunn's post-test.
